# Comparison of a Novel Bisphosphonate Prodrug and Zoledronic Acid in the Induction of Cytotoxicity in Human Vγ2Vδ2 T Cells

**DOI:** 10.3389/fimmu.2020.01405

**Published:** 2020-07-21

**Authors:** Daisuke Okuno, Yuki Sugiura, Noriho Sakamoto, Mohammed S. O. Tagod, Masashi Iwasaki, Shuto Noda, Akihiro Tamura, Hiroaki Senju, Yasuhiro Umeyama, Hiroyuki Yamaguchi, Makoto Suematsu, Craig T. Morita, Yoshimasa Tanaka, Hiroshi Mukae

**Affiliations:** ^1^Department of Respiratory Medicine, Graduate School of Biomedical Sciences, Nagasaki University, Nagasaki, Japan; ^2^Department of Biochemistry, Keio University School of Medicine, Tokyo, Japan; ^3^Center for Medical Innovation, Nagasaki University, Nagasaki, Japan; ^4^Center for Innovation in Immunoregulative Technology and Therapeutics, Graduate School of Medicine, Kyoto University, Kyoto, Japan; ^5^Department of Internal Medicine and the Interdisciplinary Graduate Program in Immunology, University of Iowa Carver College of Medicine, Veterans Affairs Health Care System, Iowa City, IA, United States

**Keywords:** bisphosphonate, cytotoxicity, mass spectroscopy, prodrug, Vγ2Vδ2 T cells

## Abstract

Increasing attention has been paid to human γδ T cells expressing Vγ2Vδ2 T cell receptor (also termed Vγ9Vδ2) in the field of cancer immunotherapy. We have previously demonstrated that a novel bisphosphonate prodrug, tetrakis-pivaloyloxymethyl 2-(thiazole-2-ylamino)ethylidene-1,1-bisphosphonate (PTA), efficiently expands peripheral blood Vγ2Vδ2 T cells to purities up to 95–99% in 10–11 days. In the present study, we first examined the effect of PTA on farnesyl diphosphate synthase (FDPS) using liquid chromatography mass spectrometry (LC-MS) to analyze the mechanism underlying the PTA-mediated expansion of Vγ2Vδ2 T cells. We find that the prodrug induced the accumulation of both isopentenyl diphosphate (IPP) and dimethylallyl diphosphate (DMAPP), direct upstream metabolites of FDPS. This indicates that not only IPP but also DMAPP plays an important role in PTA-mediated stimulation of Vγ2Vδ2 T cells. We next analyzed TCR-independent cytotoxicity of Vγ2Vδ2 T cells. When human lung cancer cell lines were challenged by Vγ2Vδ2 T cells, no detectable cytotoxicity was observed in 40 min. The lung cancer cell lines were, however, significantly killed by Vγ2Vδ2 T cells after 4–16 h in an effector-to-target ratio-dependent manner, demonstrating that Vγ2Vδ2 T cell-based cell therapy required a large number of cells and longer time when tumor cells were not sensitized. By contrast, pulsing tumor cell lines with 10–30 nM of PTA induced significant lysis of tumor cells by Vγ2Vδ2 T cells even in 40 min. Similar levels of cytotoxicity were elicited by ZOL at concentrations of 100–300 μM, which were much higher than blood levels of ZOL after infusion (1–2 μM), suggesting that standard 4 mg infusion of ZOL was not enough to sensitize lung cancer cells in clinical settings. In addition, Vγ2Vδ2 T cells secreted interferon-γ (IFN-γ) when challenged by lung cancer cell lines pulsed with PTA in a dose-dependent manner. Taken together, PTA could be utilized for both expansion of Vγ2Vδ2 T cells *ex vivo* and sensitization of tumor cells *in vivo* in Vγ2Vδ2 T cell-based cancer immunotherapy. For use in patients, further studies on drug delivery are essential because of the hydrophobic nature of the prodrug.

## Introduction

Cancer is the leading cause of deaths in developed countries. Despite recent successes in cancer immunotherapy harnessing programmed death-1 (PD-1) (1–5) and cytotoxic T lymphocyte-associated protein-4 (CTLA-4) immune checkpoint inhibitors (6), significant limitations exist for the antibody-based immunotherapies. It is thus imperative to develop additional approaches to increase the efficacy of cancer treatments. Adoptive transfer of T cells expressing T cell receptors (TCRs) that recognize tumor cells is one such strategy that shows promise (7, 8).

TCR is a membrane-anchored heterodimeric protein consisting of either α and β or γ and δ chains expressed as part of a complex with cluster of differentiation 3 (CD3). Most αβ T cells expressing α and β TCR chains recognize antigenic peptides in the context of the major histocompatibility complex (MHC) class I or class II molecules with the help of CD4 or CD8 co-receptors, whereas other αβ T cell subsets respond to lipid antigens and vitamin B precursors bound to monomorphic MHC class I-related proteins, such as CD1 family members and MR1 (9). By contrast, the mechanism underlying the antigen recognition by γδ T cells expressing γ and δ chains remains unclear. In humans, most circulating γδ T cells express Vγ2Vδ2 (also termed Vγ9Vδ2) and recognize foreign phosphoantigens (pAgs) like (*E*)-4-hydroxy-3-methylbut-2-enyl diphosphate (HMBPP) derived from pathogenic microbes (10–12) and self pAgs like isopentenyl diphosphate (IPP) (13–15). Recently, it has been demonstrated that pAgs bind to the intracellular B30.2 domain of butyrophilin (BTN) 3A1 (16–24) and the interaction between pAgs and B30.2 is sensed by Vγ2Vδ2 TCR in a BTN2A1-dependent manner (25). However, the precise mode of recognition by Vγ2Vδ2 T cells of the BTN complex has not been fully elucidated (26, 27).

Because HMBPP and IPP are pyrophosphomonoesters that can be readily hydrolyzed by esterases, nitrogen-containing bisphosphonates (N-BPs) are often utilized as stimulators for Vγ2Vδ2 T cells (28–30). N-BPs such as pamidronate (PAM) and zoledronic acid (ZOL) inhibit farnesyl diphosphate synthase (FDPS) in the mevalonate pathway (31–33), resulting in the accumulation of upstream metabolites that are recognized by Vγ2Vδ2 T cells in the context of BTN2A1 and BTN3A1. Although, ZOL is one of the most potent inhibitors of FDPS, its membrane permeability is limited because it is negatively charged. We thus previously designed and synthesized a series of N-BP prodrugs and demonstrated that treatment of antigen-presenting cells with tetrakis-pivaloyloxymethyl 2-(thiazole-2-ylamino)ethylidene-1,1-bisphosphonate (PTA) inhibited intracellular FDPS, leading to the deprivation of geranylgeranylated Rap1A, and efficiently expanded peripheral blood Vγ2Vδ2 T cells (34–37).

In the present study, we examined the mechanism by which PTA induced the stimulation of Vγ2Vδ2 T cells using mass spectrometry and determined the TCR-independent, natural (NK)-like cellular cytotoxicity, and TCR-dependent cellular cytotoxicity of PTA-expanded Vγ2Vδ2 T cells against lung cancer cells.

## Materials and Methods

### Mass Spectrometry

Tetrakis-pivaloyloxymethyl 2-(thiazole-2-ylamino)ethylidene-1,1-bisphosphonate (PTA, Techno Suzuta Co., Ltd., Heiwa-machi, Nagasaki, Japan) was dissolved in deoxymethyl sulfoxide (Nacalai Tesque, Inc., Nakagyo-ku, Kyoto, Japan) at a concentration of 1 mM and the stock solution was stored at −80°C until used. Raji Burkitt's lymphoma cells were obtained from the Japanese Collection of Research Bioresources Cell Bank (JCRB) of the National Institutes of Biomedical Innovation, Health, and Nutrition, Sennan, Osaka, Japan. Raji cells were grown in RPMI1640 medium (Merck & Co., Inc., Kenilworth, NJ), containing 10% fetal calf serum (FCS, Merck & Co., Inc.), 10^−5^ M 2-mercaptoethanol (Wako Pure Chemical Industries, Ltd., Chuo-ku, Osaka, Japan), 100 μg/ml streptomycin (Meiji Seika Pharma Co., Ltd., Chuo-ku, Tokyo, Japan), 100 U/ml of penicillin (Meiji Seika Pharma Co., Ltd.) (complete RPMI1640 medium) in 75 cm^2^ flasks (Corning Inc., Corning, NY). The cells (1 × 10^6^ cells) were treated with 0, 50, 100, 200, or 500 nM PTA in 1 ml of the complete RPMI1640 medium in 15 ml conical tubes (AGC Techno Glass Co., Ltd., Haibara, Shizuoka, Japan) at 37°C with 5% CO_2_ for 2 h. The cell suspensions were centrifuged at 600 × g at 4°C for 5 min. After the supernatants were removed, the cell pellets were dispersed by tapping and resuspended in 5 ml of cold Dulbecco's phosphate buffered saline (-) (PBS, Nissui Pharmaceutical Co., Ltd., Taito-ku, Tokyo, Japan). The cell pellets were washed two more times with cold PBS and placed in liquid nitrogen and stored at −80°C.

For the analysis of IPP and DMAPP in cell lysates, anionic metabolites were measured using an Orbitrap-type MS (Q-Exactive Focus; Thermo Fisher Scientific) connected to a high-performance ion-chromatography (IC) system (ICS-5000+; Thermo Fisher Scientific) that enables highly selective and sensitive metabolite quantification due to IC-separation and the Fourier-transform MS principle (38). The IC was equipped with an anion electrolytic suppressor (Thermo Scientific Dionex AERS 500; Thermo Fisher Scientific) to convert the potassium hydroxide gradient into pure water before the sample entered the mass spectrometer. Separation was performed using a Thermo Scientific Dionex IonPac AS11-HC, with a 4-μm particle-size column. The IC flow rate of 0.25 ml/min was supplemented post-column with 0.18 ml/min makeup flow of MeOH. The potassium hydroxide gradient conditions for IC separation were as follows: from 1 to 100 mM (0–40 min), 100 mM (40–50 min), and 1 mM (50.1–60 min), at a column temperature of 30°C. The Q-Exactive Focus mass spectrometer was operated under an ESI negative mode for all detections. Full mass scan (*m/z* 70–900) was used at a resolution of 70,000. The automatic gain control target was set at 3 × 10^6^ ions, and the maximum ion injection time was 100 ms. Source ionization parameters were optimized with a spray voltage of 3 kV, and other parameters were as follows: transfer temperature of 320°C, S-Lens level of 50, heater temperature of 300°C, Sheath gas at 36, and Aux gas at 10.

### Preparation of PBMC

Peripheral blood samples were obtained from healthy adult volunteers and lung cancer patients after approval of the Institutional Review Board of Nagasaki University Hospital and with written informed consent. All protocols were performed in accordance with the Guidelines and Regulations of Nagasaki University Hospital. The blood samples were treated with 1/100 volume of heparin sodium (Mochida Pharmaceutical., Co., Ltd., Shinjuku-ku, Tokyo, Japan) and diluted with an equal volume of PBS. The diluted blood (20 ml) was loaded on 20 ml of Ficoll-Paque^TM^ PLUS (GE Healthcare BioSciences AB, Uppsala, Sweden) in a 50 ml conical tube (Corning Inc.), which was centrifuged at 600 × g at room temperature for 30 min. The fluffy layer was collected into a 50 ml conical tube and diluted with 2.5 volumes of PBS. The diluted peripheral blood mononuclear cells (PBMC) were centrifuged at 900 × g at 4°C for 10 min and the supernatant was removed. The cell pellets were dispersed by tapping and resuspended in PBS in a 15 ml conical tube, which was centrifuged at 600 × g at 4°C for 5 min. After the supernatant was removed, the cell pellets were dispersed by tapping and resuspended in 7 ml of Yssel's medium (39), consisting of Iscove's modified Dulbecco's medium (Thermo Fisher Scientific, Waltham, MA), supplemented with 10% human AB serum (Cosmo Bio Co., Ltd., Koto-ku, Tokyo, Japan), 3.6 × 10^−2^ M NaHCO_3_ (Nacalai Tesque Inc.), 3.3 × 10^−5^ M 2-aminoethanol (Nacalai Tesque Inc.), 40 mg/l transferrin apo form (Nacalai Tesque Inc.), 5 mg/l human recombinant insulin (Merck & Co., Inc.), 2 mg/l linoleic acid (Merck & Co., Inc.), 2 mg/l oleic acid (Merck & Co., Inc.), 2 mg/ml palmitic acid (Merck & Co., Inc.), 100 μg/ml streptomycin, 100 U/ml of penicillin or RPMI1640 medium.

### Expansion of Vγ2Vδ2 T Cells

To 1.5 ml of PBMC (1–2.5 × 10^6^ cells/ml of Yssel's medium) in a well of a 24-well plate (Corning Inc.) was added 1.5 μl of 1 mM PTA stock solution to give a final concentration of 1 μM. The cells were incubated at 37°C with 5% CO_2_ for 24 h, to which was added interleukin-2 (IL-2, Shionogi Pharmaceutical Co., Ltd., Chuo-ku, Osaka, Japan) to give a concentration of 100 U/ml. After incubation at 37°C with 5% CO_2_ for one more day, the medium was replaced with Yssel's medium containing 100 U/ml IL-2. On day 2 through day 5, 100 U/ml of IL-2 was added to the medium and Vγ2Vδ2 cells were expanded. On day 6, 1.5 ml of Yssel's medium was added to the well. After being mixed well with a pipet, 1.5 ml of the cell suspension was transferred to another well. On day 7, the medium was replaced with the complete RPMI1640 medium plus 100 U/ml IL-2 and Vγ2Vδ2 T cells were expanded by day 11 and stored in liquid nitrogen until used. Cells were observed under a microscope (Nikon Corp., Minato-ku, Tokyo, Japan) on day 5 and the proportion of Vδ2 cells was determined by flow cytometry on days 0, 8, and 11 as described below. IFN-γ production was determined on day 2 as described below.

### Flow Cytometric Analysis

PBMC and PTA-expanded cells (2 × 10^5^ cells) were dispensed into a 96-well round bottom plate (Corning Inc.) and incubated with 50 μl of PBS containing 2% FCS and monoclonal antibodies (mAbs), including fluorescein isothiocyanate (FITC)-conjugated anti-TCR Vδ2 mAb (BD Biosciences, San Diego, CA) or anti-CD27 mAb (BioLegend, San Diego, CA) and phycoerythrin (PE)-conjugated anti-CD3, CD86, CD94, or CD161 mAbs (BD Biosciences), or anti-CD25 mAb (Tombo Biosciences, Co., Ltd., Kobe, Hyogo, Japan), or anti-CD45RO, CD69, NKG2D, DNAM-1, TRAIL, Fas-L, CD56, HLA-DR, HLA-DQ, or CD45RA mAbs (BioLegend), on ice for 15 min. To the wells were added 200 μl of PBS containing 2% FCS and the plate was centrifuged at 600 × g at 4°C for 2 min. After the supernatants were removed, the cell pellets were dispersed by vortexing and resuspended in 200 μl of PBS/2% FCS. The cells were washed two more times with 200 μl of PBS/2% FCS and resuspended in 400 μl of PBS/2% FCS. The cells were analyzed using a FACSCalibur flow cytometer (Becton Dickenson, Franklin Lakes, NJ) and the cell population was visualized using FlowJo ver. 10 (FlowJo LLC, Ashland, OR).

### Enzyme-Linked Immunosorbent Assay for IFN-γ

For determination of IFN-γ production from peripheral blood Vγ2Vδ2 T cells in response to PTA or ZOL, PBMC suspensions (4.35 × 10^5^ cells in 100 μl of Yssel's medium) were placed in a 96-well flat-bottom plate (Corning Inc.), to which was added 100 μl each of PTA at concentrations of 1, 3, 10, 30, 100, 300, or 1,000 nM, or zoledronic acid (ZOL, Novartis International AG, Basel, Switzerland) at concentrations of 10, 30, 100, 300, 1 μM, 3 μM, or 10 μM. The plate was incubated at 37°C with 5% CO_2_ for 24 h. To each well was added IL-2 at a final concentration of 100 U/ml. After additional 24 h incubation, the cell suspensions were mixed and the plate was centrifuged at 600 × g at 4°C for 2 min. The supernatants were transferred into a 96-well round bottom plate and placed at −80°C for 16 h. The samples were thawed and interferon-γ (IFN-γ) levels were determined by enzyme-linked immunosorbent assay (ELISA, Peprotech, Rocky Hill, NJ) according to the manufacturer's instruction.

For determination of IFN-γ production from PTA-expanded Vγ2Vδ2 T cells in response to PTA or ZOL, PBMC derived from a healthy adult volunteer was stimulated with 1 μM PTA for 11 days and the PTA-expanded Vγ2Vδ2 T cells were frozen as described above. PC-9 human lung adenocarcinoma was obtained from RIKEN BioResource Research Center (Tsukuba, Ibaraki, Japan), PC-6 human lung small cell carcinoma from Immuno-Biological Laboratories, Fujioka, Gunma, Japan), and H1975 human lung adenocarcinoma and H520 human lung squamous cell carcinoma from American Type Culture Collection (Manassas, VA). The human lung cancer cell lines were grown in 30 ml of the complete RPMI1640 medium at 37°C with 5% CO_2_ in 75 cm^2^ flasks. After the culture supernatants were removed and the cells were washed with 10 ml PBS, 2 ml each of 0.25 w/v% trypsin/1 mM ethylenediaminetetraacetic acid (EDTA) (Thermo Fisher Scientific) was added to the flasks, which were placed at 37°C with 5% CO_2_ for 2 min. To the flasks was added 10 ml each of the complete RPMI1640 medium and the cell suspensions were transferred into 15 ml conical tubes. After being centrifuged at 600 × g at 4°C for 5 min, the supernatants were removed and the cell pellets were dispersed by tapping. The cells were resuspended in 5 ml of the complete RPMI1640 medium, washed twice, and resuspended in the complete RPMI1640 medium. The cell suspensions (1.4 × 10^6^ cells/ml) were treated with PTA at final concentrations of 15.625, 31.25, 62.5, 125, 250, 500, or 1,000 nM, or with ZOL at final concentrations of 15.625, 31.25, 62.5, 125, 250, 500, or 1,000 μM at 37°C with 5% CO_2_ for 2 h. The cells were washed three times with 5 ml of the complete RPMI1640 medium and resuspended in 350 μl of the complete RPMI1640 medium. The tumor cell suspensions (4 × 10^5^ cells/100 μl) were dispensed into a 96-well round bottom plate, containing PTA-expanded Vγ2Vδ2 T cells (4 × 10^5^ cells/100 μl). The plate was incubated at 37°C with 5% CO_2_ for 16 h. Then, the cell suspensions were mixed and the plate was centrifuged at 600 × g at 4°C for 2 min. The supernatants were transferred into a 96-well round bottom plate and placed at −80°C for 16 h. The samples were thawed and interferon-γ (IFN-γ) levels were determined by ELISA according to the manufacturer's instruction.

### Luminescence-Based Cytotoxicity Assay

For determination of NK-like activity of PTA-expanded Vγ2Vδ2 T cells, PC-9, PC-6, H1975, and H520 human lung cancer cell suspensions (2 × 10^4^ cells/200 μl) were dispensed into a 96-well flat bottom plate, which was incubated at 37°C with 5% CO_2_ for 16 h. After the culture supernatants were aspirated, PTA-expanded Vγ2Vδ2 T cells were added to each well at effector-to-target (E/T) ratios of 0.3125:1, 0.625:1, 1.25:1, 2.5:1, 5:1, 10:1, 20:1, 40:1, or 80:1, and incubated at 37°C with 5% CO_2_ for 4 or 16 h. Then, the culture supernatants were aspirated and the wells were gently washed three times with 200 μl of the complete RPMI1640 medium. To the wells was added 100 μl each of CellTiterGlo® Reagent (PerkinElmer Inc., Waltham, MA), and the cell lysates were transferred into a 96-well optiplate (PerkinElmer Inc.). Luminescence was measured through an ARVO multi-plate reader (PerkinElmer Inc.). All measurements were performed in triplicate.

For determination of cellular cytotoxicity of PTA-expanded Vγ2Vδ2 T cells against PTA- or ZOL-treated PC-9, PC-6, H1975, and H520, the human lung cancer cell suspensions (2 × 10^4^ cells/200 μl) were dispensed into a 96-well flat bottom plate, which was incubated at 37°C with 5% CO_2_ for 16 h. After the culture supernatants were aspirated, 200 μl of a serially-diluted PTA was added to each well in triplicate at concentrations of 0, 0.78125, 1.5625, 3.125, 6.25, 12.5, 25, 50, or 100 nM, or a serially-diluted ZOL at final concentrations of 0, 7.8125, 15.625, 31.25, 62.5, 125, 250, 500, or 1,000 μM. The plate was incubated at 37°C with 5% CO_2_ for 2 h. After the supernatants were aspirated, 200 μl of PTA-expanded Vγ2Vδ2 T cells (3 × 10^5^ cells) were added to each well. The plate was incubated at 37°C with 5% CO_2_ for 4 or 16 h. Then, the culture supernatants were aspirated and the wells were gently washed three times with 200 μl of the complete RPMI1640 medium. To the wells was added 100 μl each of CellTiterGlo Reagent® (PerkinElmer Inc.), and the cell lysates were transferred into a 96-well optiplate (PerkinElmer Inc.). Luminescence was measured through an ARVO multi-plate reader (PerkinElmer Inc.).

### Time-Resolved Fluorescence-Based Cytotoxicity Assay

Vγ2Vδ2 T cell-mediated cellular cytotoxicity was determined using a non-radioactive cellular cytotoxicity assay kit (Techno Suzuta Co., Ltd.). PC-9, PC-6, H1975, and H520 human cancer cell lines (1 × 10^6^ cells/ml) in 15 ml conical tubes were treated with 0, 3, 10, 30, 100, 300, or 1,000 nM of PTA, or 0, 3, 10, 30, 100, 300, or 1,000 μM of ZOL at 37°C with 5% CO_2_ for 2 h and then pulsed with 2.5 μl of 10 mM bis(butyryloxymethyl) 4′-(hydroxymethyl)-2,2′:6′,2″-terpyridine-6,6″-dicarboxylate (BM-HT, Techno Suzuta Co., Ltd.) at 37°C with 5% CO_2_ for 15 min. During the incubation, BM-HT was hydrolyzed by intracellular esterases to give 4′-(hydroxymethyl)-2,2′:6′,2″-terpyridine-6,6″-dicarboxylate (HT) (40). To the conical tubes was added 5 ml of the complete RPMI140 medium and the tubes were centrifuged at 600 × g at 4°C for 5 min. After the supernatants were removed, the cell pellets were dispersed by tapping and resuspended in 5 ml of the complete RPMI1640 medium. The cells were washed two more times and resuspended in 20 ml of the complete RPMI1640 medium. The tumor cell suspensions (5 × 10^3^ cells/100 μl) were dispensed into a 96-well round bottom plate, to which were added 4 × 10^5^ Vγ2Vδ2 T cells/100 μl at an E/T ratio of 80:1. The plate was centrifuged at 200 × g at ambient temperature for 2 min and then incubated at 37°C with 5% CO_2_ for 40 min. Detergent (Techno Suzuta Co., Ltd.) at a final concentration of 5 × 10^−5^ M was added to wells for the determination of the maximum release. After the cell suspensions were mixed, the plate was centrifuged at 600 × g for 2 min and the supernatants (25 μl each) were removed to a new 96-well round bottom plate containing 250 μl of europium (Eu) solution (Techno Suzuta Co., Ltd.). After the Eu/HT complex solution was mixed, 200 μl samples were transferred to a 96-well optical plate (Thermo Fisher Scientific Inc.). Time-resolved fluorescence was measured through an ARVO multi-plate reader (PerkinElmer Inc.). All measurements were performed in triplicate. Specific lysis (%) was calculated as 100 × [experimental release (counts) – spontaneous release (counts)]/[maximum release (counts) – spontaneous release (counts)].

### Intracellular Staining for IFN-γ

PC-9 human lung cancer cells (1 × 10^6^ cells/ml) were treated with 1 ml of the complete RPMI1640 medium or the medium containing 1 μM PTA or with 1 mM ZOL at 37°C with 5% CO_2_ for 2 h. The cells were washed three times with 5 ml of the complete RPMI1640 medium and resuspended in 100 μl of the complete RPMI1640 medium. The tumor cell suspensions (5 × 10^5^ cells/50 μl) were dispensed into a 96-well round bottom plate, containing PTA-expanded Vγ2Vδ2 T cells (5 × 10^5^ cells/50 μl). The plate was incubated at 37°C with 5% CO_2_. After 2 h, brefeldin A (Merck & Co., Inc.) was added to each well at a final concentration of 10 μg/ml and the plate was incubated at 37°C with 5% CO_2_ for 2 more hours. Then, the cell suspensions were mixed and the plate was centrifuged at 600 × g at 4°C for 2 min. After the supernatants were removed, the plate was vortexed, to which was added 200 μl of PBS/2% FCS. The cells were washed two more times with 200 μl of PBS/2% FCS. Then, the cells were stained with FITC-conjugated anti-TCR Vδ2 mAb in 50 μl of PBS/2% FCS. After incubation on ice for 15 min, the cells were washed four times with PBS and fixed with 200 μl of 1% paraformaldehyde in PBS. After incubation at ambient temperature for 15 min, the plate was centrifuged at 600 × g at 4°C for 2 min. After the supernatants were removed, the plate was vortexed, to which was added 200 μl of PBS/2% FCS/0.5% saponin (Wako Pure Chemical Industries, Ltd.)/0.1% sodium azide (Merck & Co. Inc.) or Tween 20 (Nacalai Tesque). After incubation at ambient temperature for 30 min, the cells were stained with PE-conjugated anti-IFN-γ mAb (BD Biosciences) in 50 μl of PBS/2% FCS/0.5% saponin/0.1% sodium azide. After 15 min, the cells were washed three times with 200 μl of PBS/2% FCS/0.5% saponin/0.1% sodium azide and examined for intracellular IFN-γ using a FACSCalibur flow cytometer and the cell population was visualized using FlowJo ver. 10. For intracellular staining of IFN-γ in PBMC, freshly isolated PBMC were stimulated with 1 μM of PTA for 2 days and examined for the production of IFN-γ as described above.

## Results

### Intracellular Accumulation of IPP and DMAPP in PTA-Treated Target Cells

**Figure 1:**
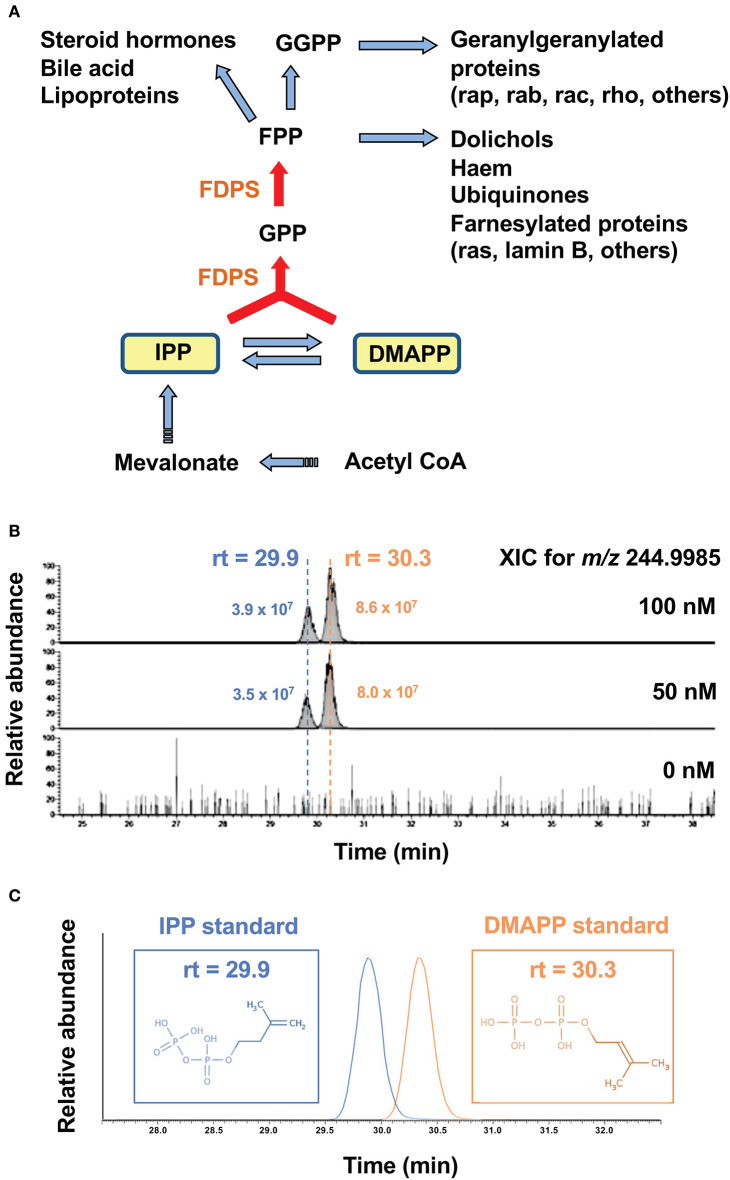
Accumulation of IPP and DMAPP in target cells after treatment with PTA. **(A)** The mevalonate pathway and the metabolites in the upstream of FDPS. Isoprenoid metabolites are synthesized from acetyl- CoA via mevalonate through IPP and DMAPP. By the action of isomerase, IPP is converted into DMAPP. FDPS, a target of PTA, catalyzes the synthesis of GPP from IPP and DMAPP, and that of FPP from GPP and IPP. Metabolites in the direct upstream of FDPS are IPP and DMAPP. **(B)** Mass spectrometric analysis of IPP and DMAPP in target cells after treatment with PTA. After treatment of Raji Burkitt's lymphoma cells with PTA (0, 50, 100 nM) for 2 h, the samples were analyzed through LC-MS and the extracted ion chromatograms (XIC) for *m/z* = 244.9985 were depicted. **(C)** Identification of IPP and DMAPP using standard compounds. The retention time for the standard IPP was 29.9 min and that for standard DMAPP was 30.3 min.

**Figure 2:**
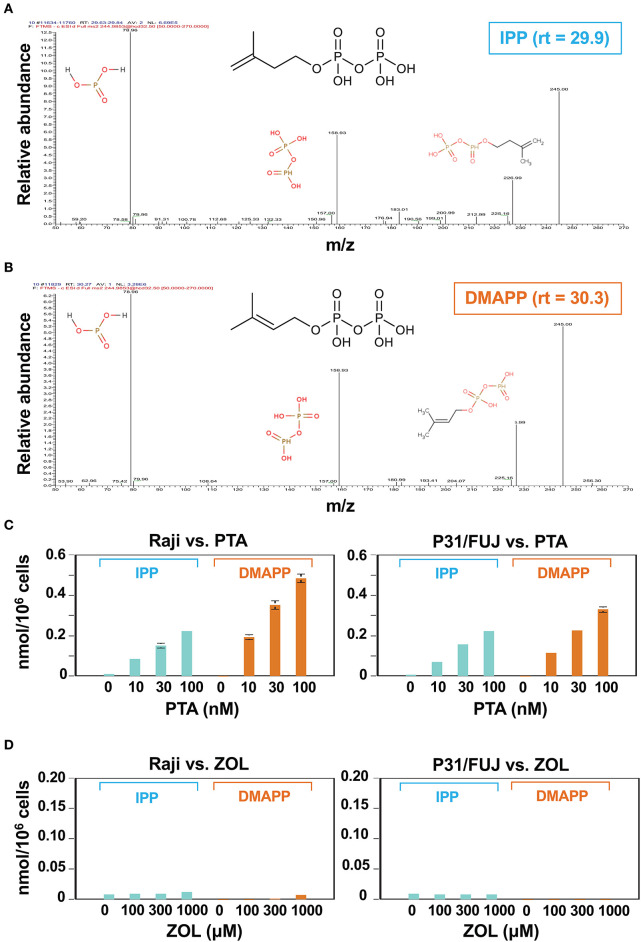
Identification and quantitative analysis of IPP and DMAPP. Daughter ion analyses of IPP **(A)** and DMAPP **(B)**. The molecular species eluted at the retention time of 29.9 and 30.3 were analyzed using IC-ESI-MS (Q-Exactive Focus; Thermo Fisher Scientific, Bremen, Germany). Molecular identification by fragmentation patterns was conducted with referring METLIN database (https://metlin.scripps.edu). Quantitative analysis of IPP and DMAPP in Raji Burkitt's lymphoma cells and P31/FUJ monocytic cells treated with PTA **(C)** or ZOL **(D)**. Cell lines (10^6^ cells each) were treated with 1 ml of a half-log serial dilution of PTA or ZOL at 37°C for 2 h and then IPP and DMAPP (nmol/10^6^ cells) were quantified using the respective standard controls.

Nitrogen-containing bisphosphonates (B-BPs) inhibit farnesyl diphosphate synthase (FDPS) as illustrated in Figure 1A. Whereas, two metabolites, isopentenyl diphosphate (IPP), and dimethylallyl diphosphate (DMAPP) exist in the direct upstream of FDPS, attention has been paid only to IPP, because IPP and DMAPP are structural isomers and it has been difficult to isolate and identify these two molecular species on mass spectrometry (MS). We introduced a novel liquid chromatography-mass spectrometry (LC-MS) system and attempted to separate these isomers on LC-MS. After Raji Burkitt's lymphoma cells were treated with tetrakis-pivaloyloxymethyl 2-(thiazole-2-ylamino) ethylidene-1,1-bisphosphonate (PTA), an N-BP prodrug, the cell lysates were examined for IPP and DMAPP. As shown in Figure 1B, both IPP and DMAPP were markedly increased after treatment of the cells with 50 or 100 nM of PTA for 2 h in the extracted ion chromatogram (XIC) for *m/z* = 244.9985. Standard IPP was eluted at the retention time of 29.9 min and DMAPP at 30.3 min (Figure 1C), confirming their structural identity, which was further corroborated by daughter ion analysis (Figures 2A,B).

In order to examine the dose-dependent effect of PTA on the intracellular accumulation of IPP and DMAPP, Raji cells (1 × 10^6^ cells) were treated with a half-log serial dilution of PTA at 37°C for 2 h and the intracellular IPP and DMAPP concentrations were quantified through LC-MS with synthetic IPP and DMAPP being used as references and expressed as nmoles/10^6^ cells. As illustrated in the left panel of Figure 2C, both IPP and DMAPP accumulated in the cells in a PTA dose-dependent manner: 0.010 ± 0.001 nmole at 0 nM, 0.084 ± 0.003 nmole at 10 nM, 0.152 ± 0.012 nmole at 30 nM, 0.222 ± 0.005 nmole at 100 nM for IPP and 0.000 ± 0.000 nmole at 0 nM, 0193 ± 0.011 nmole at 10 nM, 0.352 ± 0.020 nmole at 30 nM, 0.485 ± 0.022 nmole at 100 nM for DMAPP. The concentrations of DMAPP in Raji cells were consistently higher than those of IPP at any concentrations of PTA. When the P31/FUJ monocytic cell line was examined for IPP and DMAPP accumulation, essentially the same results were obtained: 0.009 ± 0.000 nmole at 0 nM, 0.072 ± 0.004 nmole at 10 nM, 0.158 ± 0.006 nmole at 30 nM, 0.222 ± 0.007 nmole at 100 nM for IPP, and 0.000 ± 0.000 nmole at 0 nM, 0.115 ± 0.006 nmole at 10 nM, 0.226 ± 0.002 nmole at 30 nM, 0.330 ± 0.014 nmole at 100 nM for DMAPP as depicted in the right panel of Figure 2C. In PTA-treated P31/FUJ cells, the concentrations of DMAPP were higher than those of IPP at any PTA concentrations, as observed in Raji cells. Interestingly, zoledronic acid (ZOL), one of the most potent commercially available N-BPs, induced a low level of IPP and DMAPP in both cell lines even at a concentration of as high as 1,000 μM within 2 h of pulsing (Figure 2D). Although only a low level of DMAPP was observed in Raji cells after ZOL treatment for 2 h, the accumulation was dependent on ZOL concentrations: 0.000 ± 0.000 nmole at 0 μM, 0.001 ± 0.000 nmole at 100 μM, 0.002 ± 0.000 nmole at 300 μM, 0.07 ± 0.000 nmole at 1,000 μM. These results clearly demonstrated that PTA induced intracellular accumulation of IPP and DMAPP more efficiently than ZOL.

### Expansion by PTA of Vγ2Vδ2 T Cells Derived From Healthy Adults and Lung Cancer Patients

Because intracellular accumulation of IPP and DMAPP by PTA leads to the recognition by Vγ2Vδ2 T cells, we next attempted to expand Vγ2Vδ2 T cells. Before implementing the expansion using PTA, we first examined the effect of media on the expansion of Vγ2Vδ2 T cells by ZOL. We compared RPMI1640 medium, one of the most frequently-used media, with Yssel's medium (39) used for the expansion of human natural killer (NK) cells and killer αβ T cells. After PBMC were stimulated with ZOL and expanded in the presence of IL-2, significant cell clustering was observed under a microscope as shown in Figure S1A. Regarding the initial phase of stimulation, RPMI1640 medium induced more pronounced responses in PBMC than Yssel's medium. When it comes to the proportion of Vγ2δV2 T cells, the initial percentage of Vδ2 T cells was 0.94% among the lymphocyte gate of PBMC. After expansion by ZOL in RPMI1640 medium, the proportion of Vδ2 T cells was increased up to 39.3% on day 6 and 42.8% on day 7. In case of Yssel's medium, the proportion was 43.0% on day 6 and 65.8% on day 7, respectively (Figure S1B), indicating that YM medium supported the specific expansion of Vγ2Vδ2 T cells. After expansion for 11 days, the total number of Vδ2 T cells in Yssel's medium was greater than that in RPMI1640 medium (Figure S1C). Taken together, Yssel's medium was superior to RPMI1640 medium for the specific expansion of Vγ2Vδ2 T cells.

**Figure 3:**
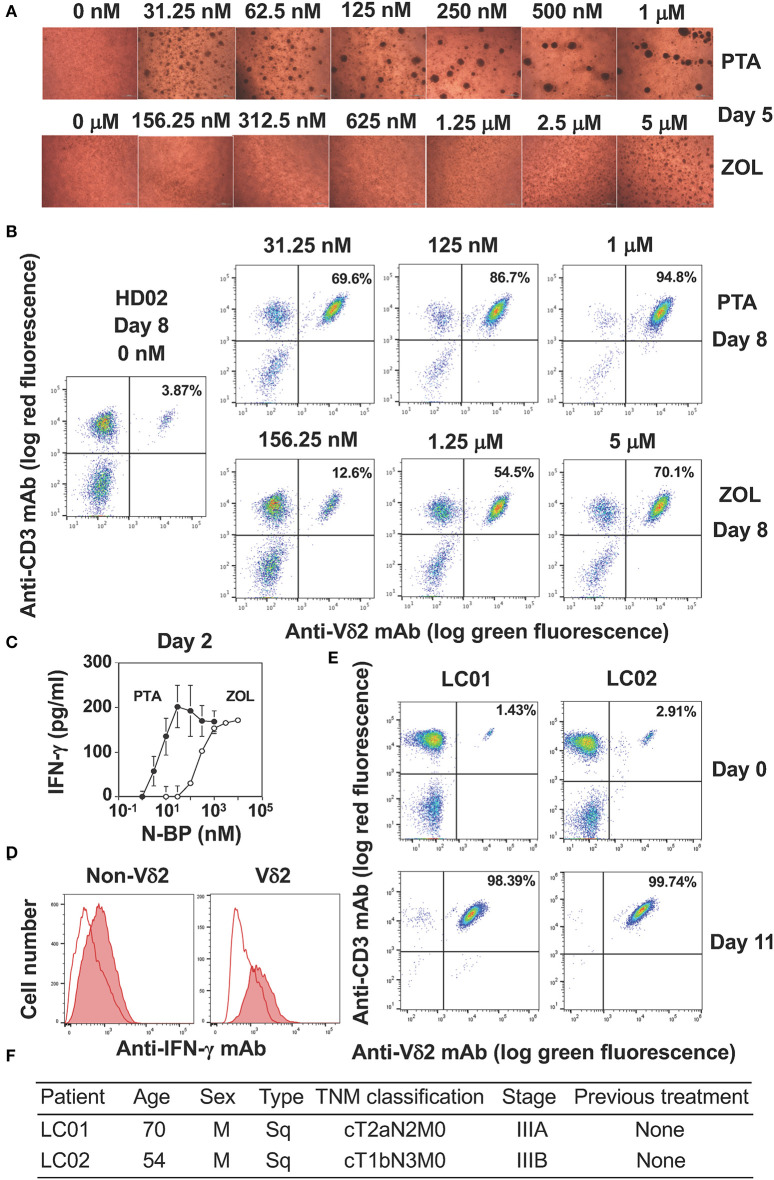
Comparison of PTA and ZOL in the expansion of peripheral blood Vγ2Vδ2 T cells. **(A)** Microscopic observation of PBMC from a healthy adult stimulated with PTA or ZOL. PBMC derived from a healthy donor (HD02) were stimulated with a serial dilution of PTA or ZOL and the cell clustering was observed under a microscope on day 5. **(B)** Flow cytometric analysis of PBMC stimulated with PTA or ZOL. PBMC derived from HD02 was stimulated as in **(A)** were examined for the expression of CD3 and Vδ2 on day 8. **(C)** IFN-γ production by Vγ2Vδ2 T cells in response to PTA or ZOL. After stimulation of PBMC with PTA or ZOL for 2 days, IFN-γ was measured through ELISA. **(D)** Intracellular staining of IFN-γ in PBMC stimulated with PTA. PBMC stimulated with PTA in **(C)** was examined for intracellular IFN-γ through flow cytometry. **(E)** Expansion of Vγ2Vδ2 T cells by PTA **(D)**. Flow cytometric analysis was performed on days 0 and 11 after stimulation of PBMC with 1 μM of PTA derived from lung cancer patients (LC01 and LC02). **(F)** Clinical characteristics of the LC01 and LC02 lung cancer patients.

We next compared PTA and ZOL in the expansion of Vγ2Vδ2 T cells in Yssel's medium. When PBMC were stimulated with a serial dilution of PTA or ZOL in the presence of IL-2, cell clustering was observed on day 5 as shown in Figure 3A. Consistent with the dose-dependency in the intracellular accumulation of IPP and DMAPP (Figure 2C), 31.25 nM of PTA was sufficient to induce cell clustering in PBMC, whereas 5 μM of ZOL was required for cell clustering on day 5. The difference between PTA and ZOL in the induction of cell clustering in PBMC was 160-fold (31.25 nM vs. 5 μM). As shown in Figure 3B, the proportions of Vγ2Vδ2 T cells on day 8 were 3.87, 12.6, 54.5, and 70.1% when stimulated with 0, 156.25 nM, 1.25 μM, and 5 μM of ZOL, and 69.6, 86.7, and 94.8% with 31.25, 125 nM, and 1 μM of PTA, respectively, demonstrating that PTA induced the specific expansion of Vγ2Vδ2 T cells to a greater degree than ZOL.

We next examined the IFN-γ production from PBMC stimulated with PTA or ZOL for 2 days. As depicted in Figure 3C, the half maximal concentration of PTA for inducing IFN-γ in PBMC was about 5 nM and that of ZOL was 200 nM, demonstrating that the difference between PTA and ZOL in the induction of IFN-γ production from peripheral blood Vγ2Vδ2 T cells was 40-fold. When PTA-treated PBMC was examined for IFN-γ by intracellular staining on flow cytometry, Vδ2^+^ T cells producing IFN-γ were detected (Figure 3D). Non-Vδ2^+^ T cells also produced a low level of IFN-γ by inflammatory cytokines possibly derived from dendritic cells, consisting with previous reports (41, 42). We then examined the effect of PTA on the expansion of Vγ2Vδ2 T cells in lung cancer patients (Figure 3E). The initial proportions of Vδ2^+^CD3^+^ T cells in peripheral blood lymphocytes derived from lung cancer patients LC01 and LC02 were 1.43 and 2.91%, respectively. After stimulation with PTA and IL-2 for 11 days, the proportions of Vδ2^+^CD3^+^ T cells were increased to 98.39 and 99.74%, respectively. The demographic data of these lung cancer patients is shown in Figure 3F. The results demonstrated that Vγ2Vδ2 T cells of lung cancer patients could be efficiently expanded by PTA, which might be used for adoptive transfer therapy.

### TCR-Independent Cellular Cytotoxicity Elicited by PTA-Expanded Vγ2Vδ2 T Cells

Human Vγ2Vδ2 T cells exhibit at least three types of cellular cytotoxicity, TCR-independent, TCR-dependent, and antibody-dependent cytotoxicity. We first analyzed cell surface markers expressed on PTA-expanded Vγ2Vδ2 T cells through flow cytometry. As shown in Figure S2, the purity of Vγ2Vδ2 T cells after expansion with PTA and IL-2 for 11 days was 99.2 and 98.5% in healthy adult volunteers HD01 and HD02, respectively. Most of the PTA-expanded Vγ2Vδ2 T cells exhibited a CD45RA^−^CD27+ phenotype and were categorized into effector memory cells. The expression of NKG2D (CD314) C-type lectin receptor was detected on almost all the cells, while another C-type lectin receptor, CD94, was expressed in a subset of Vγ2Vδ2 T cells. DNAM-1 (CD226), an immunoglobulin superfamily receptor was expressed on almost all the cells, demonstrating that NK cell-like effector functions are expected in the PTA-expanded Vγ2Vδ2 T cells, in addition to Vγ2Vδ2 TCR-dependent killer activity. It is noteworthy that the PTA-expanded Vγ2Vδ2 T cells expressed antigen-presenting cell-related molecules like CD86, HLA-DR, and HLA-DQ, suggesting that tumor antigen-presentation by Vγ2Vδ2 T cells are expected after killing of tumor cells. In the present study, therefore, we further analyzed TCR-independent and -dependent cytotoxicity of the PTA-expanded Vγ2Vδ2 T cells.

We then examined TCR-independent, NK-like activity exhibited by PTA-expanded Vγ2Vδ2 T cells against lung cancer cells. When PC-9 human lung adenocarcinoma, PC-6 human lung small cell carcinoma, H1975 human lung adenocarcinoma, and H520 human lung squamous cell carcinoma cells were challenged by Vγ2Vδ2 T cells, no explicit cytotoxicity was observed in 40 min as shown in Figure S3, indicating that Vγ2Vδ2 T cells could not kill lung cancer cells in an early phase (within 40 min) even at an effector-to-target (E/T) ratio of 80:1.

**Figure 4:**
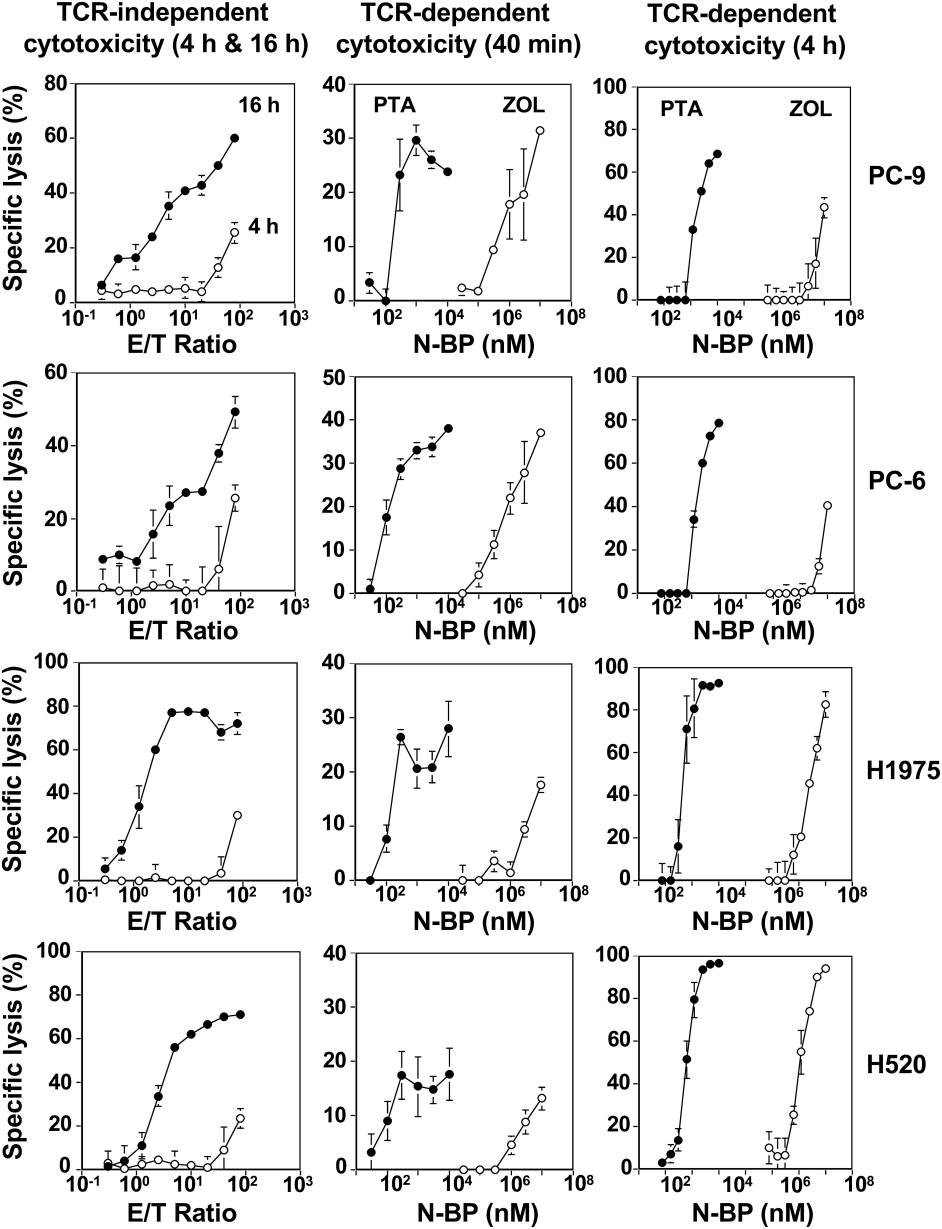
Vγ2Vδ2 T cell-mediated cytotoxicity against human lung cancer cells. (Left panels) TCR-independent cytotoxicity mediated by Vγ2Vδ2 T cells against lung cancer cell lines. PC-9 human lung adenocarcinoma, PC-6 human lung small cell carcinoma, H1975 human lung adenocarcinoma, and H520 human lung squamous cell carcinoma cells lines were challenged by PTA-expanded Vγ2Vδ2 T cells at E/T ratios of 0.3125:1, 0.625:1, 1.25:1, 2.5;1, 5;1, 10:1, 20:1, 40:1, and 80:1. After incubation for 4 h (⚬) or 16 h (•), the amount of adenosine triphosphate in viable, adherent cells were quantified by using a luciferase assay system and the specific lysis (%) was determined. (Middle panels) Early phase of TCR-dependent cellular cytotoxicity by Vγ2Vδ2 T cells against human lung cancer cells. Human lung cancer cell lines PC-9, PC-6, H1975, and H520 were pretreated with PTA (•) at concentrations of 3, 10, 30, 100, 300, and 1,000 nM, or ZOL (⚬) at concentrations of 3, 10, 30, 100, 300, and 1,000 μM and challenged by PTA-expanded Vγ2Vδ2 T cells at an E/T ratio of 80:1. After incubation for 40 min, the amount of 4′-(hydroxymethyl)-2,2′:6′,2″-terpyridine-6,6″-dicarboxylate (HT), a hydrolyzate derived from PTA by the action of intracellular esterases, released from dead target cells was quantified by harnessing Eu-based time-resolved fluorescence and the specific lysis (%) was determined. (Right panels) Later phase of TCR-dependent cellular cytotoxicity by Vγ2Vδ2 T cells against human lung cancer cells. PC-9, PC-6, H1975, and H520 human lung cancer cells (2 × 10^4^ cells) were pretreated with PTA (•) at concentrations of 0.78125, 1.5625, 3.125, 6.25, 12.5, 25, 50, or 100 nM, or ZOL (⚬) at concentrations of 7.8125, 15.625, 31.25, 62.5, 125, 250, 500, or 1,000 μM and challenged by PTA-expanded Vγ2Vδ2 T cells (3 × 10^5^ cells). After incubation for 4 h, the amount of adenosine triphosphate in viable, adherent cells were quantified by using a luciferase assay system and the specific lysis (%) was determined.

When the incubation was extended to 4 h, 20–30% of specific lysis was observed in all the four lung cancer cell lines at an E/T ratio of 80:1. Further extension of the culture to 16 h gave more extensive cellular cytotoxicity even at an E/T ratio of 10:1 as shown in the left panels of Figure 4. It is thus likely that Vγ2Vδ2 T cells require relatively long time to kill lung cancer cells when TCR is not involved in the recognition.

### TCR-Dependent Killing of Lung Cancer Cells by Vγ2Vδ2 T Cells

We next compared PTA and ZOL in the TCR-dependent killing of lung cancer cells by Vγ2Vδ2 T cells. When PTA- or ZOL-pulsed lung cancer cell lines were challenged by Vγ2Vδ2 T cells for 40 min at an E/T ratio of 80:1, the specific lysis rate attained to around 30% in all the lung cancer cell lines as shown in the middle panels of Figure 4. The concentrations required for the half maximal specific lysis rates were 10–30 nM for PTA and 100–300 μM for ZOL, with the difference between the two compounds in the sensitization of tumor cells being ~10,000-fold.

When the incubation was extended to 4 h, more than 50% of PTA- or ZOL-pulsed lung cancer cells were killed by Vγ2Vδ2 T cells at an E/T ratio of 15:1, with the drug concentrations required for the half-maximal specific lysis rates being essentially the same as those for 40 min as shown in the right panels of Figure 4. Further prolongation of the incubation time to 16 h resulted in the maximal specific lysis rate of 80% or greater, and the drug concentrations required for the half-maximal specific rates were essentially the same as those for 40 min and 4 h (Figure S4).

### IFN-γ Production From Vγ2Vδ2 T Cells in Response to PTA-Pulsed Lung Cancer Cells

**Figure 5:**
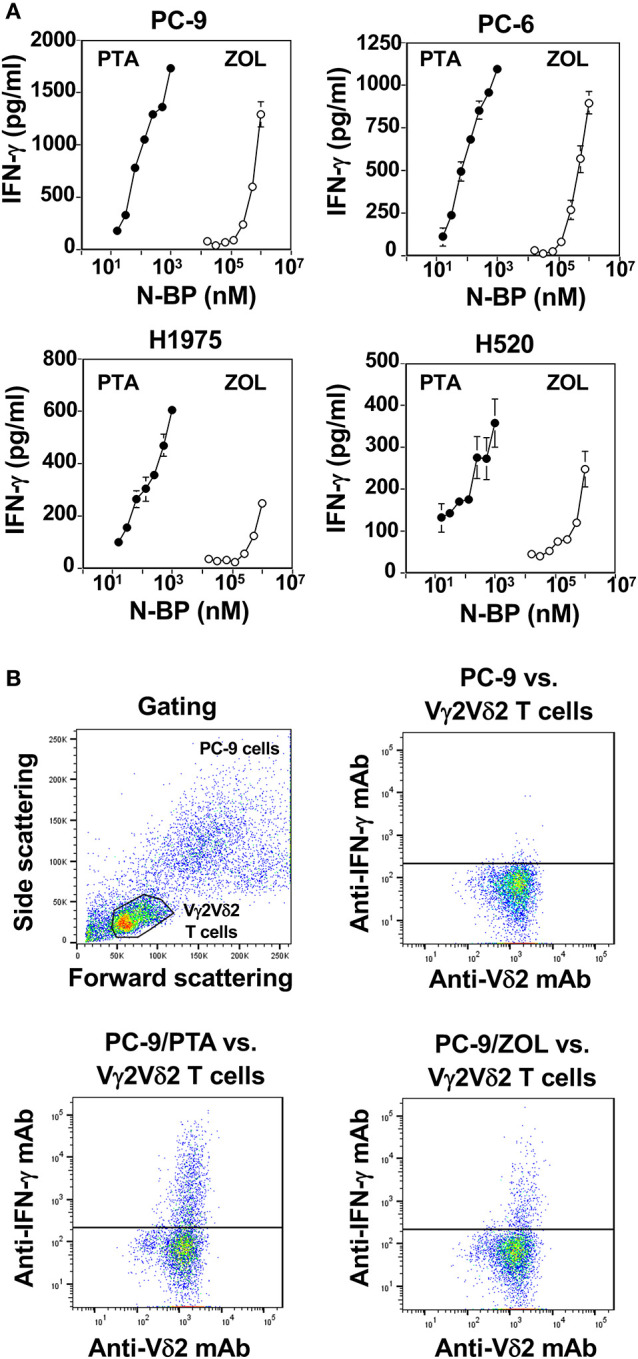
Comparison of PTA and ZOL in the induction of IFN-γ from Vγ2Vδ2 T cells. **(A)** Determination of IFN-γ produced by Vγ2Vδ2 T cells in response to lung cancer cell lines pulsed with PTA or ZOL. Human lung cancer cells, PC-9, PC-6, H1975, and H520 (4 × 10^5^ cells), pretreated with PTA (•) at concentrations of 15.625, 31.25, 62.5, 125, 250, 500, or 1,000 nM, or ZOL (⚬) at concentrations of 7.8125, 15.625, 31.25, 62.5, 125, 250, 500, or 1,000 μM were challenged by PTA-expanded Vγ2Vδ2 T cells (4 × 10^5^ cells). After incubation for 16 h, the culture supernatants were examined for IFN-γ levels through ELISA. **(B)** Intracellular staining of IFN-γ in Vγ2Vδ2 T cells in response to human lung cancer cells pretreated with PTA or ZOL. PC-9 human lung cancer cells (1 × 10^6^ cells/ml) were treated with 1 ml of the complete RPMI1640 medium or the medium containing 1 μM PTA or with 1 mM ZOL at 37°C with 5% CO_2_ for 2 h. Then, the cells were examined for intracellular IFN-γ using a FACSCalibur flow cytometer and the cell population was visualized using FlowJo ver. 10.

Finally, we compared PTA and ZOL in the induction of IFN-γ in Vγ2Vδ2 T cells. When PTA- or ZOL-pulsed lung cancer cells were incubated with Vγ2Vδ2 T cells, IFN-γ was secreted from Vγ2Vδ2 T cells in a compound dose-dependent manner, in which the drug concentrations required for the half-maximal IFN-γ production were 10–30 nM for PTA and 100–300 μM for ZOL (Figure 5A), consistent with the results observed in cellular cytotoxicity assay. The secretion of IFN-γ from Vγ2Vδ2 T cells was confirmed by intracellular staining of IFN-γ as shown in Figure 5B, in which the amount of IFN-γ secreted from Vγ2Vδ2 T cells in response to PTA-pulsed lung cancer cells was more than that to ZOL-pulsed target cells.

## Discussion

Since the success of CTLA-4 and PD-1 immune checkpoint inhibitors, increasing attention has been paid to cancer immunotherapy (1, 5). Adoptive transfer of immune effector cells is one of the most promising strategies, because the critical phase of cancer immunotherapy is the killing of tumor cells by immune effector cells. Whereas, a variety of immune effector cells are involved in the eradication of tumor cells, it is generally difficult to prepare a large number of functionally robust immune effector cells for adoptive immunotherapy. For instance, NK cells occupy 10–20% of peripheral blood mononuclear cells. When NK cells are stimulated, nearly 100-fold expansion is observed in 10 days (43). Prolonged culture, however, leads to the accumulation of functionally altered or exhausted phenotype of NK cells, resulting in the limitation of NK cell-based immunotherapy (44). Regarding CD8^+^ αβ T cells, the proportion of tumor antigen-specific CD8^+^ αβ T cells in tumor-infiltrating T lymphocytes (TILs) is only 10% and it is difficult to expand a large number of functional tumor antigen-specific CD8^+^ T cells for adoptive immunotherapy (45). By contrast, it is relatively easy to expand Vγ2Vδ2 T cells using pyrophosphomonoester pAgs or N-BPs for adoptive immunotherapy. Previously, we synthesized PTA, a novel N-BP prodrug, and demonstrated that PTA induced an efficient expansion of Vγ2Vδ2 T cells. Because the mechanism underlying the responses of Vγ2Vδ2 T cells to PTA was, however, not fully elucidated, we first examined the effect of PTA on the activity of FDPS in tumor cells as a model system, a possible target of PTA, at a molecular level.

Since the discovery that metabolites in the mevalonate pathway in human and other animal cells and the 2-*C*-methyl-D-erythritol 4-phosphate/1-deoxy-D-xylulose 5-phosphate (MEP/DOXP) non-mevalonate pathway in microbial pathogens, attention has been paid to IPP as an endogenous ligand and HMBPP as a foreign PAg (46). After it was found that PAM and ZOL inhibited FDPS in the mevalonate and non-mevalonate pathways, N-BPs, especially ZOL, have been used for the expansion of Vγ2Vδ2 T cells in the laboratory and for clinical trials, because the inhibition of FDPS by N-BPs leads to the intracellular accumulation of IPP, which is the metabolite upstream of FDPS and has activity in stimulating Vγ2Vδ2 T cells in a BTN2A1/3A1-dependent manner (31–33, 47, 48).

Whereas, both IPP and DMAPP are the direct upstream metabolites of FDPS, DMAPP has not been studied extensively, because it is difficult to distinguish DMAPP from IPP in N-BP-pulsed target cells on MS analyses. We recently developed a methodology to isolate and identify the two metabolites. In this study, we applied this strategy to detect IPP and DMAPP in tumor cells pulsed with PTA, an N-BP prodrug, which by itself has no inhibitory activity for FDPS. PTA is a hydrophobic prodrug compound and readily permeates target cell membranes. Once PTA is incorporated into target cells, intracellular esterases hydrolyze the prodrug to give (thiazole-2-ylamino)ethylidene-1,1-bisphosphonate (TA), a hydrophilic compound that directly inhibits FDPS. It is difficult for TA to permeate the membrane, resulting in the accumulation of the compound within the target cells (36).

Based on the present LC-MS study, it is clear that both IPP and DMAPP accumulate in tumor cells pulsed with PTA and that the amount of DMAPP in the PTA-pulsed cells is ~2-fold greater than that of IPP in Raji Burkitt's lymphoma cells and ~1.5-fold in P31/FUJ monocytic cells. Since the Vγ2Vδ2 T cell-stimulating activity of IPP is 2 to 3-fold higher than that of DMAPP (15), the physiological significance of the intracellularly accumulated DMAPP in the tumor sensitization for Vγ2Vδ2 T cells is equivalent to that of IPP. Taken together, it is most likely that both IPP and DMAPP are equally functional and important in stimulating Vγ2Vδ2 T cells when antigen-presenting cells are pulsed with PTA.

For developing adoptive transfer of Vγ2Vδ2 T cells, it is imperative to establish an efficient protocol for preparing Vγ2Vδ2 T cells, in which the number and purity are the two major issues. We examined the effect of medium on the expansion of Vγ2Vδ2 T cells. Since ZOL is commonly used to expand Vγ2Vδ2 T cells in the laboratory, we used ZOL as a stimulant. In order to establish or maintain human NK cells and CD8 killer T cells, Yssel's medium is often used (39). We thus compared Yssel's medium with one of the most commonly used RPMI1640 medium in the expansion of Vγ2Vδ2 T cells. Based on the present study, stimulation of PBMC with ZOL/IL-2 in RPMI1640 medium resulted in non-specific, stimulatory effects on lymphocytes including NK cells and αβ T cells at the initial phase of expansion until day 4–5, but the non-specific stimulation failed to sustain longer expansion of Vγ2Vδ2 T cells. In contrast, non-specific stimulation of lymphocytes was limited in Yssel's medium at the initial phase and specific expansion of Vγ2Vδ2 T cells was observed until day 11, resulting in the high number and purity of Vγ2Vδ2 T cells.

It was previously shown that negatively-charged N-BPs taken up by monocytes or dendritic cells through fluid-phase endocytosis inhibited FDPS, resulting in the depletion of the downstream metabolites including geranylgeranyl diphosphate (49). One of the metabolites derived from geranylgeranyl diphosphate is geranylgeraniol that might stabilize proaspase-1 (50). Procaspase-1 is a precursor of caspase-1 that converts pro-interleukin-1 (IL-1β) into mature IL-1β and pro-IL-18 into mature IL-18. Since IL-1β induces inflammation and IL-18 enhances the inflammation, the depletion of geranylgeraniol might induce inflammation. Furthermore, it was demonstrated that soluble factors such as IL-18 derived from dendritic cells pulsed with ZOL enhanced the production of IFN-γ in non-Vδ2 cells like NK cells when PBMC were treated with ZOL/IL-2 (41, 42). In addition, the inhibition of FDPS by PTA results in the accumulation of IPP and DMAPP, followed by the condensation of IPP and adenosine monophosphate (AMP) to yield triphosphoric acid 1-adenosin-5′-yl ester 3-(3-methylbut-3-enyl) ester (APPPI) (51). It was demonstrated that mitochondrial and lysosomal membrane permeabilization was inhibited by APPPI, resulting in cell death and subsequent inflammation. The present study indicates that Yssel's medium somehow prevents the inflammation caused by IL-1β/IL-18/IFN-γ and APPPI and sustains the specific expansion of Vγ2Vδ2 T cells triggered by accumulation of IPP and DMAPP in a BTN2A1/3A1-dependent manner.

We then compared PTA and ZOL in the stimulation and expansion of peripheral blood Vγ2Vδ2 T cells. The half-maximal concentration of PTA required for stimulating circulating Vγ2Vδ2 T cells was ~30 nM, whereas that of ZOL was ~5 μM, demonstrating that PTA was about 100-fold more effective in stimulating peripheral blood Vγ2Vδ2 T cells than ZOL. In stimulating peripheral blood Vγ2Vδ2 T cells with ZOL, the major subsets of antigen-presenting cells are adherent cells, such as monocytes and dendritic cells (52). These cells can take up many small molecules including positively- or negatively-charged compounds through fluid-phase endocytosis (49, 53). Although ZOL is negatively-charged under a physiological condition, the drug can be taken up by monocytes and dendritic cells at a clinical concentration of 2 μM (54). Based on the findings in the present study, it is reasonable to use ZOL for the expansion of Vγ2Vδ2 T cells, whereas PTA is more convenient and efficient to obtain a large number of highly purified Vγ2Vδ2 T cells for adoptive transfer therapy for cancer and possibly for infectious diseases, because the number and purity of PTA-expanded Vγ2Vδ2 T cells are higher than those of ZOL-stimulated Vγ2Vδ2 T cells. Phenotypic analysis of the PTA-expanded Vγ2Vδ2 T cells revealed that most of them exhibited effector memory phenotype (55) and expressed NK cell receptors like NKG2D and DNAM-1. As previously reported, antigen-presenting cell-related molecules such as CD86, HLA-DQ, and HLA-DR were also expressed, suggesting that Vγ2Vδ2 T cells might serve as antigen-presenting cells as well as immune effector cells.

When N-BP-expanded Vγ2Vδ2 T cells are used for adoptive cell therapy for cancer, TCR-dependent recognition of tumor cells is not anticipated, except for particular malignant cells, such as Dauid Burkitt's lymphoma and RPMI8226 multiple myeloma cells, based on previous studies (56). In this study, we examined the TCR-independent, NK-like cellular cytotoxicity of Vγ2Vδ2 T cells against lung cancer cell lines. Since human NK cells kill malignant cells including K562 erythrocytoma cells in 40 min (43), we employed the same 40 min protocol for the killing by Vγ2Vδ2 T cells of lung cancer cell lines, including PC-9 human lung adenocarcinoma, PC-6 human lung small cell carcinoma, H1975 human lung adenocarcinoma, and H520 human lung squamous cell carcinoma cells. Based on the time-resolved fluorescence assay, the lung cancer cell lines were resistant to NK-like activity of Vγ2Vδ2 T cells in a relatively short period of incubation.

Vγ2Vδ2 T cells-mediated NK-like cytotoxicity against lung cancer cells became apparent after 4 h of incubation at an E/T ratio of 80:1, based on luminescence-based assay. Further extension of the incubation increased the specific lysis rates even at an E/T ratio of 2.5:1. These results demonstrate that Vγ2Vδ2 T cells can kill lung cancer cells in an NK-like manner, whereas a higher E/T ratio and a longer incubation time are required for the manifestation of the Vγ2Vδ2 TCR-independent, NK-like cellular cytotoxicity, compared to those for conventional human NK cells against K562 cells.

It was previously demonstrated that commercially available N-BPs such as PAM and ZOL could sensitize not only monocytes and dendritic cells, but also tumor cells (52, 57). In this assay system, N-BP-sensitized tumor cells were killed by Vγ2Vδ2 T cells in a TCR-dependent manner (56). We then compared PTA and ZOL in the sensitization of lung cancer cells, PC-9, PC-6, H1975, and H520, for Vγ2Vδ2 T cells. In the 40-min assay, the concentrations required for the half-maximal specific lysis rates were 10–30 nM for PTA in all the lung cancer cell lines. By contrast, 100–300 μM of ZOL was required for the same level of cellular cytotoxicity, with the difference between PTA and ZOL being ~10,000-fold. Further extension of incubation time to 4 or 16 h also resulted in essentially the same difference between the two compounds.

It is worthy of note that 100–300 μM of ZOL is required for the sensitization of lung cancer cells for Vγ2Vδ2 T cells, which is much higher than the plasma concentration (1–2 μM) after infusion of 4 mg of ZOL (54). This finding confirms that ZOL can be used for the expansion of Vγ2Vδ2 T cells, because antigen-presenting cells like monocytes and dendritic cells can take up ZOL efficiently through fluid-phase endocytosis (49, 53). It is, however, unlikely that ZOL can sensitize lung cancer cells for Vγ2Vδ2 T cells, since the plasma ZOL concentration (1–2 μM) is not enough to fully inhibit FDPS in lung cancer cells.

By contrast, PTA readily permeates tumor cell membranes, where esterases hydrolyze the prodrug to yield an active TA that can efficiently inhibit FDPS (36). It is thus prerequisite to develop N-BP prodrugs for successful Vγ2Vδ2 T cell-based immunotherapy for lung cancer. Although pivaloyloxymethyl group-protected prodrugs of N-BPs exhibit a high level of activity for sensitizing Vγ2Vδ2 T cells, they are generally too hydrophobic and suitable drug delivery system has to be developed in the future.

## Data Availability Statement

The datasets generated for this study are available on request to the corresponding author.

## Ethics Statement

The studies involving human participants were reviewed and approved by Institutional Review Board of Nagasaki University Hospital. The patients/participants provided their written informed consent to participate in this study.

## Author Contributions

YS and YT designed the research. DO, YS, MT, MI, SN, AT, HS, YU, and YT performed the experiments. DO, YS, NS, HY, MS, CM, YT, and HM prepared the manuscript. YT and HM supervised the overall project. All authors discussed the results and commented on the manuscript.

## Conflict of Interest

YT is a co-inventor of Japanese Patent 2014-257451 on the development of the method to expand γδ T cells using PTA, a novel bisphosphonate prodrug and of Japanese Patent 2014-73475 on the development of a non-radioactive cellular cytotoxicity assay using BM-HT, a precursor of a novel Eu^3+^ chelate-forming compound. The remaining authors declare that the research was conducted in the absence of any commercial or financial relationships that could be construed as a potential conflict of interest.
