# Supplementary material for: Comparison of a Novel Bisphosphonate Prodrug and Zoledronic Acid in the Induction of Cytotoxicity in Human Vγ2Vδ2 T Cells
Source: Front Immunol. 2020 Jul 21;11:1405. doi: 10.3389/fimmu.2020.01405 (PMC7385076; doi:10.3389/fimmu.2020.01405)
Supplement: Supplementary file 1 [file Presentation_1.pdf]

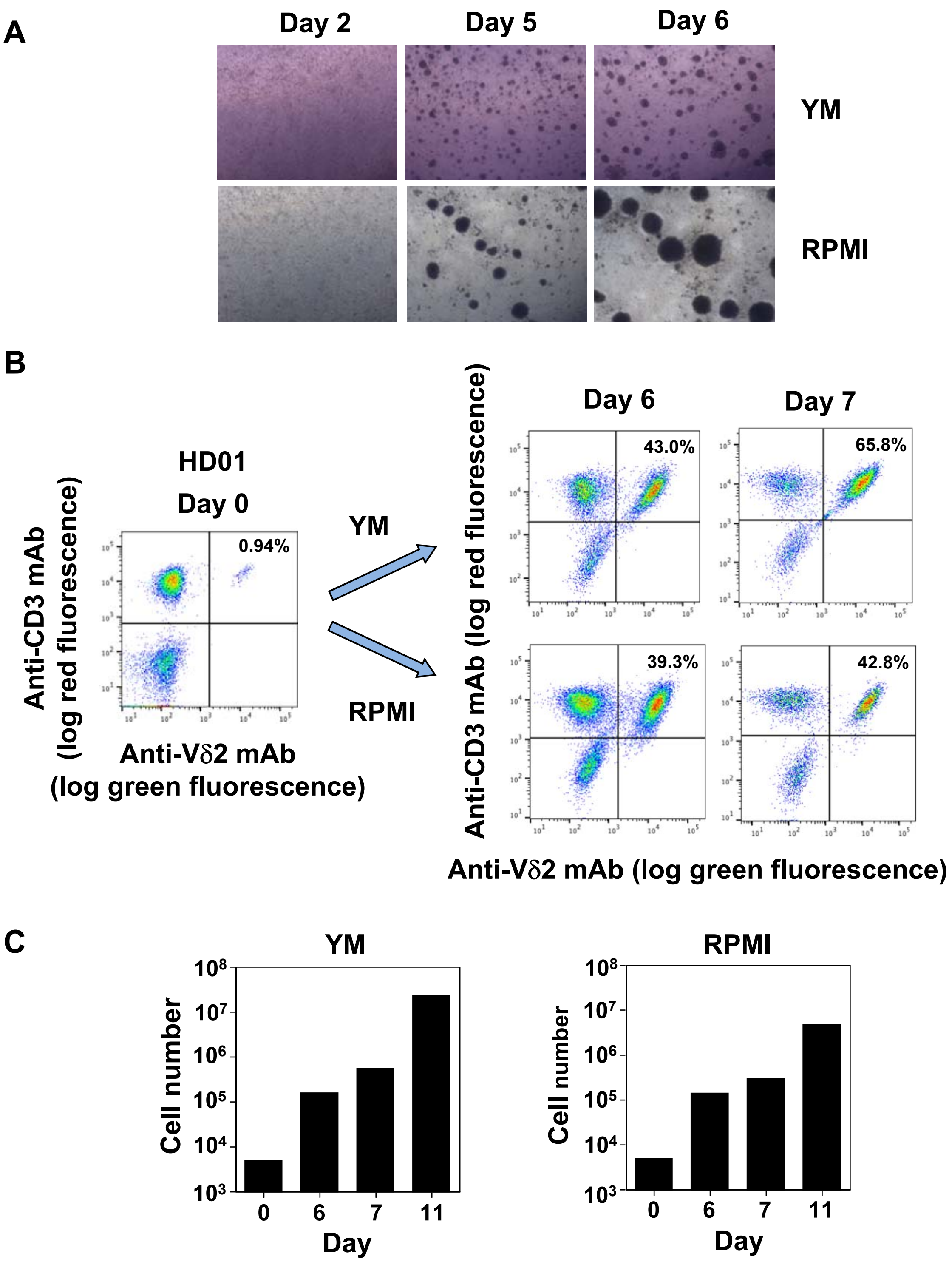

Supplementary Fig. 1. Effect of culture media on the expansion of Vγ2Vδ2 T cells. (A) Microscopic images of PBMC stimulated with ZOL. PBMC derived from a healthy donor (HD1) was stimulated with 5 μM of ZOL in either Yssel's medium or RPMI1640 medium. Cell clustering was observed under a microscope on days 2, 5, and 6. **(B) Flow cytometric analysis of Vγ2Vδ2 T cells stimulated with ZOL.** Cultured cells in (A) were stained with PE-conjugated anti-CD3 mAb and FITC-conjugated anti-Vδ2 mAb on days 0, 6, and 7 and analyzed using a FACS Verse flow cytometer. **(C) Effect of culture media on the proliferation of Vγ2Vδ2 T cells.** After stimulation with ZOL in (A), the number of Vγ2Vδ2 T cells was calculated based on the cell number and flow cytometric analysis.

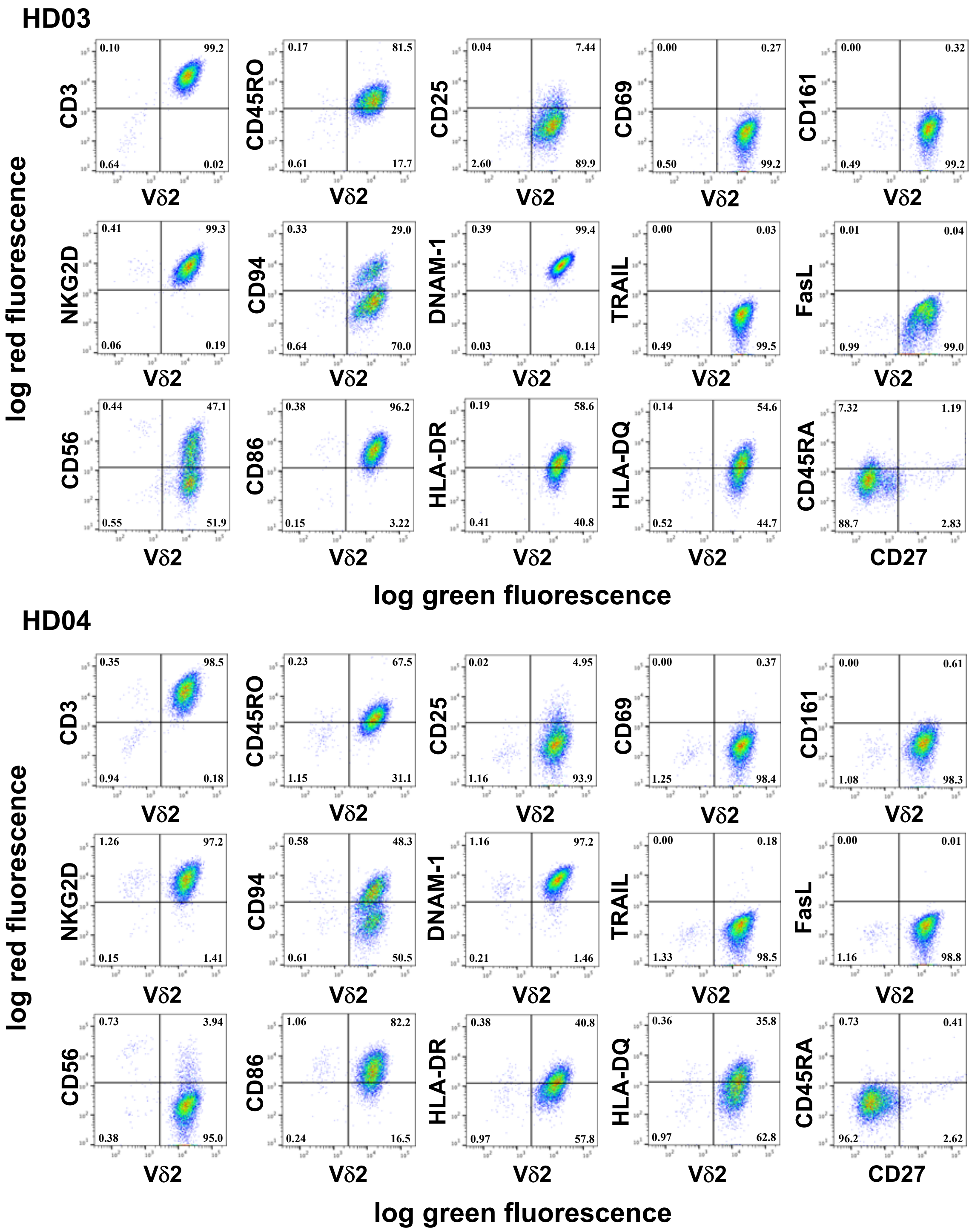

Supplementary Fig. 2. Phenotypic analysis of Vγ2Vδ2 T cells expanded with PTA. PBMC derived from two healthy donors (HD03 and HD04) were stimulated with 1 μM of PTA in Yssel's medium supplemented with human AB serum and Vγ2Vδ2 T cells were expanded in the presence of IL-2 for 11 days. The cells were stained with PE-conjugated anti-CD3, CD45RO, CD25, CD69, CD161, NKG2D, DNAM-1, CD94, TRAIL, FasL, CD56, CD86, HLA-DR, HLA-DQ, or CD45RA mAb and FITC-conjugated anti-Vδ2 or CD27 mAb.

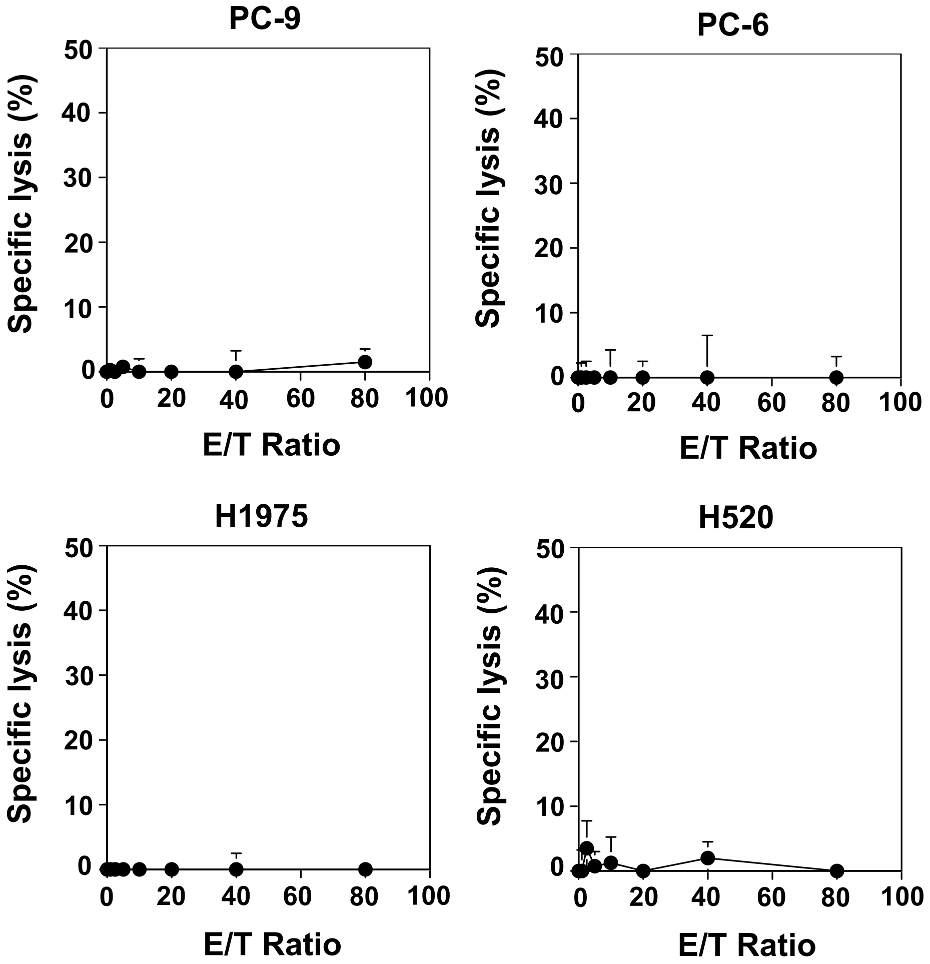

Supplementary Fig. 3. Vγ2Vδ2 T cells failed to kill human lung cancer cell lines in 40 min. PC-9, PC-6, H1975, and H520 human lung cancer cell lines (1 x 10^6 cells/ml) in 15 ml conical tubes were pulsed with 2.5 μl of BM-HT at 37°C with 5% CO_2 for 15 min. To the conical tubes was added 5 ml each of the complete RPMI140 medium and the tubes were centrifuged at 600 x g at 4°C for 5 min. After the supernatants were removed, the cell pellets were dispersed by tapping and resuspended in 5 ml of the complete RPMI1640 medium. The cells were washed two more times and resuspended in 20 ml of the complete RPMI1640 medium. The tumor cell suspensions (5 x 10^3 cells/100 μl) were dispensed into a 96-well round bottom plate, to which were added 100 μl of PTA-expanded Vγ2Vδ2 T cells at effector-to-target ratios of 0:1, 1.25:1, 2.5:1, 5:1, 10:1, 20:1, 40:1, and 80:1. The plate was centrifuged at 200 x g at ambient temperature for 2 min and then incubated at 37°C with 5% CO_2 for 40 min. Detergent was added to wells for the determination of the maximum release. After the cell suspensions were mixed, the plate was centrifuged at 600 x g for 2 min and the supernatants (25 μl each) were removed to a new 96-well round bottom plate containing 250 μl of Eu solution. After the solution was mixed, 200 μl samples were transferred to a 96-well optical plate (Thermo Fisher Scientific Inc.). Time-resolved fluorescence was measured through an ARVO multi-plate reader (PerkinElmer Inc., Waltham, MA). All measurements were performed in triplicate. Specific lysis (%) was calculated as 100 x [experimental release (counts) – spontaneous release (counts)]/[maximum release (counts) – spontaneous release (counts)].

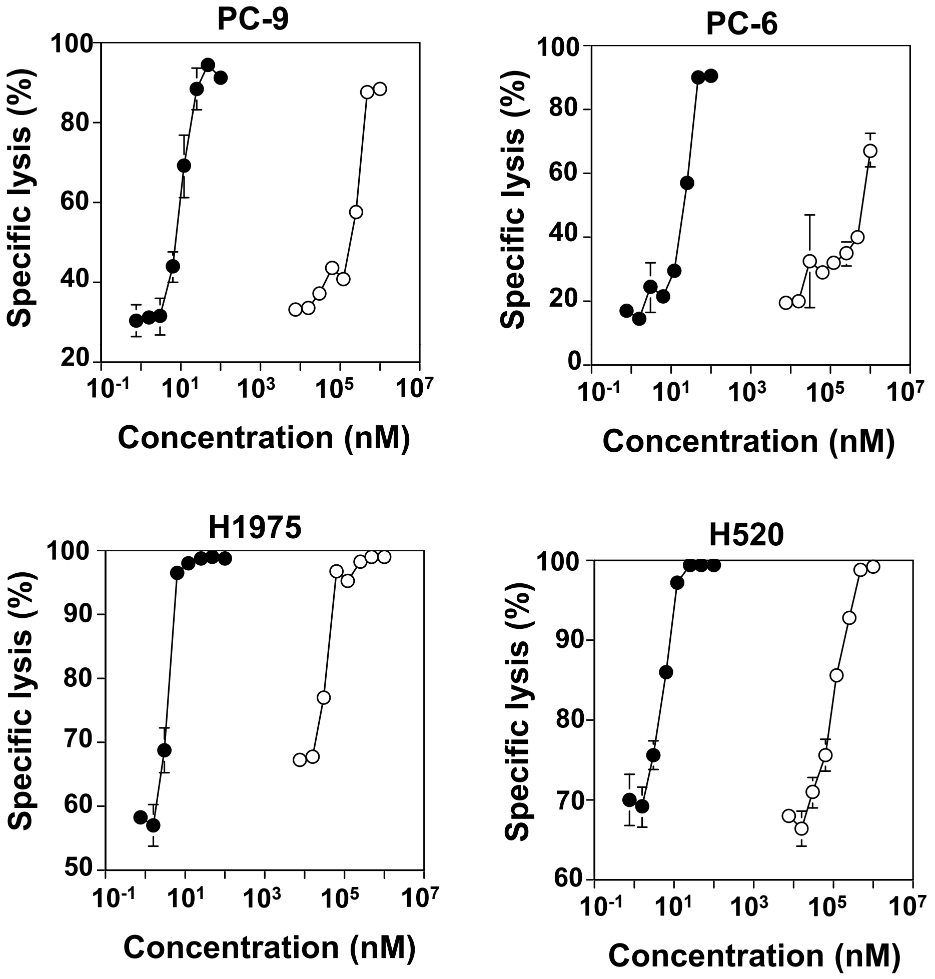

Supplementary Fig. 4. Comparison of PTA and ZOL in the sensitization of human lung cancer cells for Vγ2Vδ2 T cells. PC-9, PD-6, H1975, and H520 human lung cancer cells (2 x 10^4 cells/200 μl) were dispensed into a 96-well flat bottom plate, which was incubated at 37°C with 5% CO_2 for 16 h. After the culture supernatants were aspirated, 200 μl of a serially-diluted PTA (●) was added to each well in triplicate at concentrations of 0.78125 nM, 1.5625 nM, 3.125 nM, 6.25 nM, 12.5 nM, 25 nM, 50 nM, or 100 nM, or a serially-diluted ZOL (○) at final concentrations of 7.8125 μM, 15.625 μM, 31.25 μM, 62.5 μM, 125 μM, 250 μM, 500 μM, or 1000 μM. The plate was incubated at 37°C with 5% CO_2 for 2 h. After the supernatants were aspirated, 200 μl of PTA-expanded Vγ2Vδ2 T cells (3 x 10^5 cells) were added to each well. The plate was incubated at 37°C with 5% CO_2 for 16 h. Then, the culture supernatants were aspirated and the wells were gently washed three times with 200 μl of the complete RPMI1640 medium. To the wells was added 100 μl each of CellTiterGlo Reagent, and the cell lyzates were transferred into a 96-well optiplate. Luminescence was measured through an ARVO multi-plate reader (PerkinElmer Inc.).
